# Retrospective Analysis of Patients with Gynaecological Uterine Sarcomas in a Tertiary Hospital

**DOI:** 10.3390/jpm12020222

**Published:** 2022-02-06

**Authors:** Maria Ruiz-Minaya, Elsa Mendizabal-Vicente, Wenceslao Vasquez-Jimenez, Laura Perez-Burrel, Irene Aracil-Moreno, Carolina Agra-Pujol, Mireia Bernal-Claverol, Beatriz L. Martínez-Bernal, Mercedes Muñoz-Fernández, Melanie Morote-Gonzalez, Miguel A. Ortega, Santiago Lizarraga-Bonelli, Juan A. De Leon-Luis

**Affiliations:** 1 Department of Public and Maternal and Child Health, School of Medicine, Complutense University of Madrid, 28040 Madrid, Spain; mruiz341060@salud.madrid.org (M.R.-M.); elsa.mendizabal@salud.madrid.org (E.M.-V.); lpburrel@salud.madrid.org (L.P.-B.); irene.aracil@salud.madrid.org (I.A.-M.); mireia.bernal@salud.madrid.org (M.B.-C.); santiago.lizarraga@salud.madrid.org (S.L.-B.); jaleon@ucm.es (J.A.D.L.-L.); 2 Department of Obstetrics and Gynecology, University Hospital Gregorio Marañón, 28009 Madrid, Spain; 3 Health Research Institute Gregorio Marañón, UDMIFFA, 28009 Madrid, Spain; 4 Peritoneal Carcinomatosis, Sarcomas and Complex Pelvis Unit, General Surgery and Digestive System Service, Gregorio Marañón General Hospital, 28009 Madrid, Spain; wenceslao.vasquez@salud.madrid.org (W.V.-J.); melanie.morote@salud.madrid.org (M.M.-G.); 5 Pathological Anatomy Service, Gregorio Marañón General Hospital, 28009 Madrid, Spain; caroagra@ucm.es (C.A.-P.); blmartinezbernal@ucm.es (B.L.M.-B.); 6 Radiotherapy Oncology Service, Gregorio Marañón General Hospital, 28009 Madrid, Spain; mmunozfernandez2@salud.madrid.org; 7 Department of Medicine and Medical Specialties, Faculty of Medicine and Health Sciences, University of Alcalá, Alcalá de Henares, 28801 Madrid, Spain; 8 Ramón y Cajal Institute of Healthcare Research, 28034 Madrid, Spain

**Keywords:** uterine sarcoma, gynaecological malignancies, comparative analysis, systematic review

## Abstract

Uterine sarcomas are rare and heterogeneous malignancies accounting for 1% to 3% of all gynaecological tumours. There are many histological subtypes recognised, including leiomyosarcomas, endometrial stromal sarcoma, and uterine carcinosarcoma, although the latest has been recently discarded in this group. Despite its low incidence, these types of cancer currently entail multiple challenges, either in diagnostics or clinical management, with a poor prognosis associated. The present work aimed to complete a comparative analysis of the different histological subtypes based on the clinicopathological characteristics of our population, the therapeutic characteristics, and associated prognosis in 161 patients treated in our centre during the period between 1985 and 2020. Moreover, a systematic review grouped a total of 2211 patients with a diagnosis of uterine sarcoma from 19 articles published in 16 countries from 2002 to 2021 was performed, all with retrospective analyses. Our results showed that apart from uterine carcinosarcoma, leiomyosarcoma is the most frequent subtype of uterine sarcoma, with unique clinical, demographic, and survival parameters. To our knowledge, this is the first systematic review conducted in this field and, thus, it shows the difficulties of collecting a significant number of patients per year, a valid reason why multicentre or national registries are recommended to allow a more exhaustive analysis of this pathology.

## 1. Introduction

Uterine sarcomas are a heterogeneous group of malignant tumours that originate from the mesenchymal tissues of the uterus, for example, the endometrial stroma, the myometrium, and the connective tissue. They are rare tumours that constitute approximately 1–3% of gynaecological cancers and 3–7% of all malignant uterine tumours [1,2,3,4,5,6].

The term uterine sarcoma encompasses several histological subtypes, such as leiomyosarcoma (LMS), endometrial stromal sarcoma (ESS), and undifferentiated uterine sarcoma (UUS), and other less frequent histological subtypes, such as adenosarcoma, rhabdomyosarcoma, and malignant perivascular epithelioid cell tumour (PEComa).

In 2009, FIGO developed a new staging system for uterine sarcomas [7]. Thereafter, uterine carcinosarcoma (CS) that had been classically classified as a uterine sarcoma was considered an endometrial carcinoma with sarcomatoid differentiation and therefore was staged and treated as a high-grade endometrial carcinoma. However, due to the worse prognosis compared to that for common endometrial lesions, CSs were included in retrospective studies of uterine sarcomas after the implementation of the new system. In this study, we included CSs in the analysis because they were classified as uterine sarcomas during much of the study period.

The incidence of this type of tumour increases with age and its incidence is reported to be 0.5 to 2.1 per 100,000 women [6,8]. The typical age at which this type of tumour appears is 50 to 70 years of age but varies depending on the histological subtype [4,5,8,9,10].

Diagnosing these tumours can be challenging because there is no specific symptomatology, and differential diagnosis by imaging can be complex and difficult to perform because sometimes these tumours present an appearance very similar to that of benign uterine lesions, such as fibroids (especially LMS).

Due to the rarity of uterine sarcomas and the heterogeneity of the population, the optimal treatment remains a subject of debate. Surgery continues to be the mainstay of treatment, while radiotherapy and chemotherapy have roles as adjuvant treatments; palliative treatments are provided for metastatic or recurrent disease.

These tumours tend to metastasize and recur early; therefore, the prognosis is poor, with an overall 5-year survival rate lower than 50% [4,11,12,13,14].

The present study described 30 years of experience of a reference centre with patients diagnosed with uterine sarcoma and performed a comparative analysis of the different histological subtypes based on demographic, clinical, tumour, tumour staging, and therapeutic variables as well as on prognoses related to the survival of these patients.

## 2. Patients and Methods

### 2.1. Observational Study

A hospital-based cohort study of patients diagnosed with uterine sarcoma between January 1985 and August 2020 was conducted.

This study was carried out in a tertiary hospital in multidisciplinary collaboration with the different services that are responsible for the management of this pathology.

All patients included in our analysis had a histological diagnosis of uterine sarcoma and were treated in our centre. Cases in which it was not possible to obtain information on the treatment and evolution of the patients by reviewing the clinical history and cases in which there were doubts in the histopathological diagnosis were excluded. Specifically, based on the histological subtype, the following were included: leiomyosarcomas (LMSs), endometrial stromal sarcomas (ESSs), carcinosarcomas (CSs), and other subtypes, such as adenosarcomas and rhabdomyosarcomas.

For each patient, the following data were collected: clinicopathological characteristics—age at diagnosis, race, presence of comorbidities, menopausal status, clinical and FIGO (International Federation of Gynecology and Obstetrics) staging at diagnosis (in those sarcomas diagnosed before 2009, the FIGO 1988 staging was used; in those diagnosed between 2009 and 2018 the FIGO 2009 staging; and in those diagnosed after 2018, the FIGO 2018 staging), histological subtype; therapeutic management—primary treatment, date of surgery, type of surgical resection and approach, lymphadenectomy, gastrointestinal tract anastomosis, postsurgical residual tumour (R0), operative duration (minutes), postsurgical complications and grade III-IV severe complications according to CTACAE (NCI Common Terminology Criteria for Adverse Events), need for reintervention, length of hospital stay, perioperative death (30 days), in-hospital death (>30 days), and need for neo/adjuvant treatment (chemotherapy and/or radiotherapy); and follow-up and survival—tumour progression, recurrence, type and location of metastatic disease, overall survival (OS; defined as the period of time from uterine sarcoma surgery to the date of the last revision surgery or death), and disease-free survival (DFS; defined as the period of time after primary treatment without evidence of tumour recurrence or progression).

The data for each variable were extracted retrospectively from a review of the clinical history of each patient.

The main objective of our study was to describe the clinicopathological characteristics of our population, as well as the therapeutic characteristics, and to analyse the OS and DFS as a function of the anatomopathological type of uterine sarcoma in the patients treated in our centre during the period between 1985 and 2020.

The data obtained for the study were entered into Microsoft Office Excel, version 16.0, and the statistical analysis was performed with SPSS, version 26. Quantitative variables are presented as the mean and standard deviation (S.T), and categorical variables are presented as the number of patients and rates (%) (95% CI). For the analysis of the quantitative variables, Student’s t-test or analysis of variance (ANOVA) for independent samples was used. Regarding the qualitative variables, the comparison of proportions was performed by means of the chi-squared test. For the OS and DFS analyses, the Kaplan-Meier method was used, and the respective subtype analysis was conducted with the log rank statistical test. Differences with *p* < 0.05 were considered statistically significant.

Due to CS in the surgical-pathological stage of disease being based on new FIGO staging for the endometrial carcinoma and to distinguish this type of tumor in this study, its results were remarked in the tables.

This study was conducted following the STROBE guidelines (Strengthening the Reporting of Observational Studies in Epidemiology), and its implementation was approved by the ethics committee of our hospital and was classified by the AEMP (Spanish Agency of Medicines and Health Products) as a non-EPA study (“Non-interventional post-authorisation observational study”).

### 2.2. Systematic Review

This systematic review was conducted in accordance with the PRISMA criteria.

#### 2.2.1. Eligibility Criteria and Outcome Measures

The fundamental objective of the systematic review was to evaluate whether the latest published articles, especially those with a publication date after 2009, included uterine carcinosarcomas in the analysis.

The inclusion criteria were observational studies, multicentre studies, systematic reviews, and meta-analyses that described clinicopathological, therapeutic, and survival characteristics in patients diagnosed with uterine sarcoma.

#### 2.2.2. Information Sources and Search Strategies

The search was carried out in the electronic database PubMed/MEDLINE on 11 April 2021, using “uterine sarcoma” or a combination of the terms “sarcoma” and “uterus”. A reference database (EndNote X7, Thomson Reuters, New York, NY, USA) was used to incorporate all references.

#### 2.2.3. Study Selection and Data Extraction

The following filters were applied: humans, Spanish or English language, female sex, articles with access to the full text, and time period between 1985–2021; the following were excluded: review articles, clinical trials, books and documents.

All articles were analysed independently by two authors (MRM y JLL), and if the title and abstract did not provide useful information for the review or were irrelevant, the articles were omitted from the analysis. Disagreement between the two researchers was resolved by consensus.

The full text of the articles included in the systematic review was obtained and evaluated, and their references were also analysed to find new articles for inclusion.

Data collection was performed with a standard form and each reviewer collected the data independently and included them in the extraction sheet. Discrepancies were resolved by both authors checking the study against the form. The variables that were collected for each article were title, author, year of publication, country in which the study was developed, design and type of study (single or multicentre), total number of patients, number of patients by subtype of uterine sarcoma, mean age at diagnosis, most frequent clinical manifestation, whether uterine carcinosarcomas were included in the analysis, and OS at 5 years. 

A descriptive study of all the articles included in the study was carried out.

The statistical analysis was performed using SPSS, version 26.0, in its default configuration.

The risk of bias was evaluated to determine the adequacy of meeting the inclusion criteria.

## 3. Results

### 3.1. Observational Study

#### 3.1.1. Clinical Data

**Figure 1:**
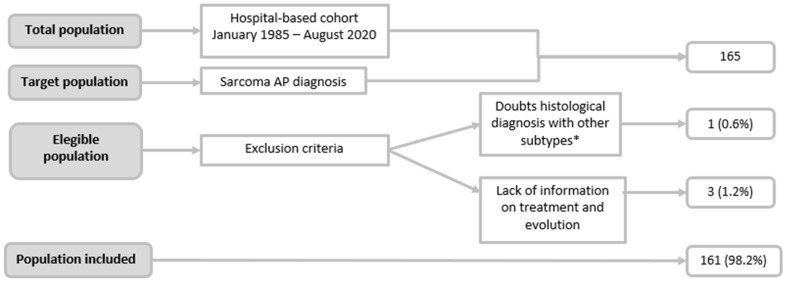
Observational Study Patient inclusion WorkFlow. * The histopathological evaluation was carried out by two anatomopathologists specialized in gynecological histopathology.

A total of 161 patients diagnosed with uterine sarcoma were included in our study. The flow chart of the included patients is shown in Figure 1, and Table 1 summarizes the clinicopathological characteristics at diagnosis.

The mean age at diagnosis was 59.5 years (range 45–73 years), and 66.6% of the patients were younger than or equal to 65 years of age at diagnosis. The lowest age at diagnosis was observed in the ESS subgroup, with a mean age at diagnosis of 55 years.

The patient distribution based on subtype was as follows: 51 with LMSs, 31 with ESSs, 71 with CSs, and six with other less frequent histological subtypes (five with adenosarcomas and one with a rhabdomyosarcoma). Among those with ESSs, 24 (77%) had high-grade lesions, and six had low-grade lesions; for one patient, the grade was unknown.

There were statistically significant differences among the four groups with respect to postmenopausal state. The percentage was lower in the LMS subgroup.

Vaginal bleeding was the most frequent clinical manifestation in 63.8% of patients. For LMSs, the most frequent form of presentation (44.23%) was incidental finding in hysterectomy specimens indicated for suspected benign pathology. A total of eight patients had a history of previous use of tamoxifen (six CS, one ESS and one others), and three had received pelvic radiation therapy (RT) prior to the appearance of uterine sarcomas (two CSs and one ESS). Three had received pelvic RT prior to the appearance of sarcoma due to rectal adenocarcinoma.

Based on FIGO staging, 62.3% of the patients were in early stages (stages I–II) at the time of diagnosis, and 37.7% had locally advanced or disseminated disease at the time of diagnosis.

#### 3.1.2. Surgical Treatment and Pathological Findings

Table 2 shows the characteristics of the treatment of patients with uterine sarcoma.

Surgery was the first line of treatment for 93.2% (150) of patients. For the remaining 11 patients, no surgical treatment was performed because the disease was in an advanced stage and because of the clinical conditions of the patients.

The diagnosis of uterine sarcoma was not suspected prior to surgery in 21.7% of patients (45% for LMSs).

A hysterectomy was performed in all patients, and in 90% of patients, bilateral adnexectomy was also performed. Lymphadenectomy was performed in the event of suspicious lymph nodes and depending on the histological type; its completion rate was significantly higher in the CS group (61.4%) than in the LMS group (8.2%) and ESS group (25.9%). It was only performed at the para-aortic level in one patient (0.66%). The intervention rate at the level of the digestive tract was 6%, with no statistically significant differences among the groups.

Complete resection was achieved in 84.6% of the patients, without differences among the histological subtypes. The rate of postoperative complications was low, 8.6%, with 4.6% being severe complications based on CTCAE. The mean length of hospital stay was 6–7 days.

#### 3.1.3. Adjuvant Therapy

A total of 53.4% of patients received adjuvant treatment (Table 3); specifically, patients diagnosed with CS most frequently received adjuvant treatment with chemotherapy, radiotherapy, or both.

For patients in whom radiotherapy was used as a complementary treatment, such therapy was more frequently administered in the form of external radiotherapy combined with brachytherapy.

#### 3.1.4. Progression and Recurrent Disease

Tumour progression is defined as the progression of the tumour without achieving temporary remission of the disease.

Tumour progression occurred in 27 patients (16.8%), with no statistically significant differences among the different subtypes. In most cases, patients were only candidates for palliative treatment. 

Recurrence is defined as the reappearance of a tumour after a disease-free period.

Sarcoma recurred in 73 patients (45.3% of cases), with no differences among the different subtypes. The most frequent form of recurrence was multiple metastases (80.8%), with no statistically significant differences observed among the different subtypes. The characteristics of relapse and treatment are shown in Table 4. Only 29 patients experienced relapse after surgical treatment, with R0 resection achieved in 86.2% of patients. The mean time from surgery to recurrence was 19 months.

The most frequent forms of presentation of disseminated disease were pulmonary, hepatic, or peritoneal dissemination (Table 5). For patients with LMSs, there was a greater tendency of pulmonary involvement (*p* < 0.01).

#### 3.1.5. Survival and Prognostic Factors

The mean follow-up period for the 161 patients was 69.4 months (range 1–142 months). Table 6 provides the last known status of the patients. At the end of the study, 75 patients had died, 72 of whom had uterine sarcoma. The mean age at death was 64 years, which was lower in the subgroup diagnosed with LMS (57 years; *p* < 0.001).

The median OS was 176 months (95% CI 106.3–245.7), and the OS rates at 5 and 10 years were 54.2 and 46.2%, respectively. The OS rate at the end of the study was 23.1%.

The median disease-free survival was 61.86 months (95% CI 34.96–88.75), and the DFS rates at 5 and 10 years were 49.7 and 43.6%, respectively. The OS and DFS data, as well as survival at 5 and 10 years, for the total cohort and for each subtype are summarized in Table 7.

**Figure 2:**
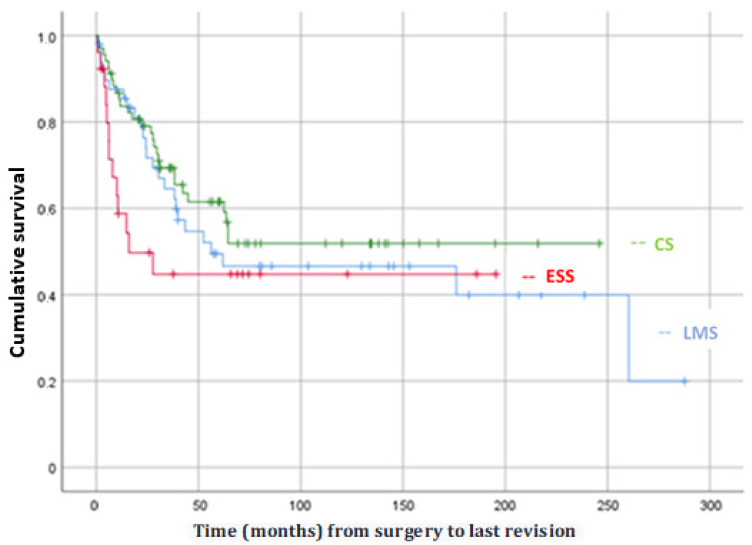
Graph of global survival by subtypes (excluding others) estimated by Kaplan-Meier curve.

No statistically significant differences were observed with respect to OS or DFS among the different histological subtypes. Analysing the data in detail, a lower OS and a lower DFS in the ESS group were striking (Figure 2). These differences are considered clinically relevant even though statistical significance was not reached.

After a differential analysis by postmenopausal status, the median OS was lower in postmenopausal patients (56.1 months, 95% CI 34.3–77.8); the difference was statistically significant (*p* < 0.04). Additionally, the median DFS was lower in postmenopausal women (42 months, 95% CI 20.4–64.4) than in premenopausal women (232 months, 95% CI 15.39–448.71) (*p* < 0.04).

Differentiating by FIGO stage at diagnosis (initial stages I–II versus advanced stages III–IV), statistically significant differences were observed in both OS (*p* < 0.01) and DFS (*p* < 0.01). The median OS was 260.3 months (95% CI 37.1–483.51) for patients at initial stages at diagnosis versus 31 months (95% CI 0.51–61.52) for patients at advanced stages (III–IV) at diagnosis, and the median DFS was 176 months (95% CI 83.4–268.6) for patients at stages I–II at diagnosis compared with 27.7 months (95% CI 14.8–40.6) for patients at stages III–IV at diagnosis.

OS and DFS were lower in the group of patients in which R0 resection was not achieved after surgery (median OS 176 months in the R0 group versus 26.7 months in the non-R0 group; DFS 103.6 months in the R0 group versus 11.6 months in the non-R0 group). These differences were statistically significant (*p* < 0.01).

### 3.2. Systematic Review

**Figure 3:**
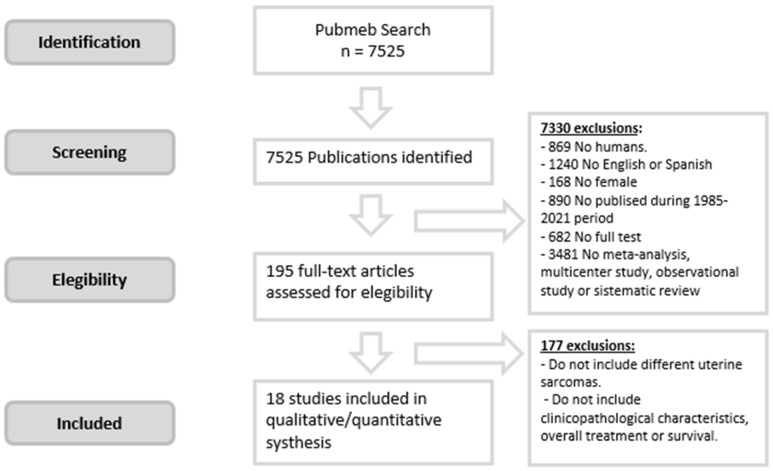
Systematic review workflow.

A total of 7525 citations were identified in the literature search. The initial selection identified a total of 7330 that did not meet the inclusion criteria after applying filters. Of a total of 195 studies that were retrieved to evaluate eligibility, 18 were selected as relevant [1,3,4,5,6,8,9,10,11,12,13,15,16,17,18,19,20,21]. Figure 3 shows a flow chart that illustrates the selection of studies.

The 18 articles included were published between 2002 and 2021 in 16 countries (Table 8). The study with the most years of follow-up (excluding ours) was that by Gao et al. [3], with 30 years of follow-up; that with the least was by Nano et al. [1], with 6 years of follow-up. The vast majority of articles were unicentric and had an average n of 116.4; the largest sample sizes corresponded to multicentre studies. Our study was a single-centre study with the largest sample size. 

Taking into account the year 2009, when the change in FIGO staging occurred, of the six articles published previous to this date, only the one by Abeler et al. [5] excluded CS. Starting in 2009, 12 studies were published, of which six did not present CS data.

The overall cumulative incidence was 6.4 cases of uterine sarcoma per year, and the average age at diagnosis was 55.8 years. The most frequent clinical manifestation at diagnosis in most studies was genital bleeding.

In general, 36.3% of these tumors were diagnosed in advanced stages (III–IV) and presented a recurrence rate of 42%.

The mean OS rate and DFS rate at 5 years were 41.4% and 36.9%, respectively.

## 4. Discussion

### 4.1. Observational Study

This study provided a general description of 35 years of experience at our centre, during which 161 patients diagnosed with uterine sarcoma were treated, for an incidence of 4–5 cases per year. This description focused on differentiating cases by histological subtype, with the most frequent being carcinosarcoma (45.3%). Statistically significant differences were found between some of the variables analysed, including postmenopausal status, advanced FIGO stage at diagnosis, and the existence of postsurgical residual tumours, which were each associated with poorer survival. The median OS was 176 months, and the DFS was 61.8 months. The analysis of survival by histological subgroup was not statistically significant, failing to identify differences that may have been clinically relevant.

Taking into account the number of patients in our series and in comparison with the various published studies, those that contributed a greater number of cases in less time were multicentre studies. Abeler et al. (2009) [5] included 419 patients with sarcomas, followed by Wais M. et al. [10] with 302 patients, the latter excluding CSs from their analysis. Regarding studies from a single centre, such as ours, Burghaus et al. [8] and Park et al. [19] performed their analyses on 143 and 127 patients with sarcomas, respectively. Although all authors agreed on the low incidence per year of this type of tumour, described between 0.5 and 2.1 per 100,000 women [1,2,3,4,5,6,8], the large number of patients included in our study was the result of 35 years during which no significant differences were observed.

Because of this long study period, CS was not excluded from our series, although in 2009, the FIGO proposed exclusion from the sarcoma group. In our study, CSs represented 45.3% of cases, followed by LMSs, which constituted 31.7%. Taking into account other published studies that included CSs, despite the change in FIGO staging, the results herein are similar to those of Eiriz et al., 44% [11], and other studies [12], lower than that reported by Sait et al., 58% [9], and higher than that reported by Gao Y. et al., 23% [3]. We do not know the substantial basis for these differences, but they may be due to differences in the mean age of the patients treated by each centre.

Excluding CS from the classification, LMS was the most frequent subtype of uterine sarcoma, constituting 57.9% of cases. These data agree with the results reported by other authors, i.e., between 40–60% of uterine sarcomas [1,2,4,5,6,8,16,17,18,19,22].

Again, taking into account the entire series and focusing on clinical variables, particularly age, herein, the mean age at diagnosis was 59.5 years, very similar to other studies [4,5,8,9,10,11]. Among the subgroups, there were significant differences, highlighting 4 years earlier in the ESS group and 3 years later in the CS and other sarcoma groups (*p* < 0.001). For the majority of the authors, both ESS and LMS present early [8,12], which in turn is related to the premenopausal status being significantly more frequent in the LMS group (*p* < 0.001). These differences in mean age were related to the frequency of associated comorbidities, being statistically more frequent in the CS group (72.6%) (*p* = 0.01). The most frequently associated medical pathology was arterial hypertension, in 33.5% of patients.

No significant differences were found among the subgroups for the history of previous pelvic RT, in agreement with that published by Benito et al. and Koivisto-Korander et al. [12,13], nor were differences found in the history of tamoxifen use.

Regarding the clinical presentation, we found significant differences among the subgroups, highlighting bleeding in the CS group (84.9%) and incidental finding in surgical specimens in the LMS group (45%), similar to those reported by Van Den Haak et al. [22] and higher than those described by Wang et al., 25% [23]. Specifically, for LMSs, a presurgical diagnosis of leiomyoma is suspected, and subsequently, the histopathological analysis reveals tumour cell growth. In addition, this subtype occurs in two out of three patients in early stages, generally as a mass confined to the uterus that can be confused with a leiomyoma.

Regarding stage at diagnosis, the differences found among the subgroups trended towards statistical significance (*p* = 0.05). A total of 62.3% were diagnosed at early stages; similar data have been reported in the literature, ranging from 56.4 to 69% [3,4,8,11,12,13,18]. In contrast, other studies, such as those by Sait et al. [9] and Kokawa et al. [21] reported higher rates of diagnosis at advanced stages (III–IV), constituting 51.6% and 52.8%, respectively. As has been described, two out of every three LMSs are diagnosed at stage I, while two out of every three of the other sarcoma subgroup are diagnosed at more advanced stages. Although more than half of patients with ESSs are diagnosed at the initial stages, in agreement with Kokawa et al. [21], we do not know why it is that up to 29% of patients are diagnosed at stage IV. This is probably because 77% of the ESSs in our sample were high grade.

Regarding the treatment variables as a function of the groups, we did not find significant differences for most of the data analysed. Surgery is the most widespread treatment for this type of tumour, and in our series, it was the first-line treatment for 93.2% of patients, with an average time from diagnosis to surgery of less than 1 month. The literature reports very similar surgical treatment rates ranging from 87.6% to 98% [3,11,12,13].

Regarding the surgical approach, 74.6% were laparotomies, and the differences among groups was significant (*p* = 0.08), with a higher percentage (85.1%) in the ESS group. Regarding the less-frequent laparoscopy route (13.3%), this approach was used the most in the CS group (18.8%). The published rates in the literature are higher than those for our sample; Koivisto-Korander et al. [13] reported 83% laparotomy, and Gao et al. [3] reported 98.7% laparotomy. These laparotomy rates may be secondary to a higher stage at diagnosis, a larger tumour size, and the greater use of laparotomy in previous decades. Although it is not the object of our study, a greater tendency to use laparoscopy was observed.

Taking into account the various surgical techniques closely related to the performance of lymphadenectomy, there were statistically significant differences among the groups. Lymphadenectomy is used less frequently for LMS (*p* < 0.01); in this histological subtype, lymphadenectomy is only indicated if pathological lymphadenopathy is observed by imaging tests or during surgery. Specifically, in the surgical techniques used for treatment, the least used was debulking (<10%), highlighting hysterectomy associated with bilateral adnexectomy in 90% of cases. A similar rate was reported by Benito et al., 93.6% [12], and a slightly higher rate was reported by Koivisto-Korander et al., 97% [13]; however, Burghaus et al. [8] reported a lower rate of bilateral adnexectomy, ranging from 65% to 74%. These variations in adnexectomy rates highlight the existing controversy regarding the possibility of preserving the ovaries [2]. Some studies report that performing bilateral adnexectomy improves the prognosis of patients with LMS and decreases the recurrence of ESS; however, other studies have not observed significant differences in OS or DFS [19]. Ovarian preservation can be considered in premenopausal women with CSs and ESSs in early stages [8]. In our study, the ovaries were preserved in only 10% of patients, with no differences among subgroups.

As described, in our series, significant differences were found in the lymphadenectomy rate (38%), a rate slightly higher than the 32.5% reported by Koivisto-Korander et al. [13]. The highest lymphadenectomy rates were ESSn in the CS subgroup (61.4%), followed by the ESS subgroup (25.9%). These high lymphadenectomy rates for CS have been reported previously: Burghaus et al. [8] reported a rate of 67%, and Gao et al. [3] reported a rate of 88.9%. This is mainly due to the higher rate of lymph node involvement that this tumour subtype presents (approximately 22%) [3,12,24]; lymphadenectomy has been part of the necessary surgical staging for this type of tumour since 2009, when it was classified as a high-grade endometrial carcinoma.

In our series, the rate of complete tumour resection was 84.6%. Other studies reported complete resection rates after surgery ranging from 72% to 87.7% [8,15,19]. This variable is closely related to the stage at diagnosis and the prognosis of patients with this type of tumour, with higher rates of incomplete resection in locally advanced disease and lower survival for patients in whom R0 surgery is not achieved.

A total of 53.4% of the patients received adjuvant treatment, with statistically significant differences among the subgroups, highlighting a greater use of adjuvant treatment for the management of patients with CS (75.3% of cases), followed by those with ESS (38.7%). The rate of adjuvant treatment use was lower than the 58.4% reported by Benito et al. [12] and 78% reported by Koivisto-Korander [13]. These differences may be primarily due to the stage at diagnosis, the persistence of postsurgical residual tumours, and the number of CSs included in the sample.

In independent analyses of the use of adjuvant RT and chemotherapy (CT) in our series, the percentages were 40.4% and 22.4%, respectively. In the literature, adjuvant RT and CT rates range from 14% to 51% and 9% to 69%, respectively [1,9,12,13]. The variability reflects the fact that there is little evidence to support the use of adjuvant RT and/or CT in any type of uterine sarcoma except in CS.

In turn, we found significant differences among the subgroups in the use of adjuvant RT, which was highest in the CS group (60.3%), followed by the ESS group (29%). In contrast, we did not find differences among subgroups in the use of adjuvant chemotherapy, but there was a greater tendency to use adjuvant chemotherapy in the CS group (31.5%), followed by the LMS group (17.6%). The rates of adjuvant RT for CS in our series were higher than those reported in the literature (48–51%) [8,13,15], and those of adjuvant CT were similar to the 35% rate observed by Burghaus et al. [8], approximately half of that reported by Durnali et al. [15] and much higher than the rate of 0% reported by Benito et al. [12]. These differences reflect the notorious controversy over the benefit of this type of treatment in recent decades.

In our study, we had a recurrence rate of 45.3%, with no statistically significant differences among the different subtypes. These tumours have a high recurrence rate; some studies report a recurrence rate of 50–70% [4,12,18]; others, such as those by Benito et al. [12] and Sait et al. [9], observed recurrence rates lower than 35% and 22%, respectively. This is also reflected in a DFS rate of less than 50%. The DFS rates reported in the literature range from 30% to 36% [19]. In our study, the DFS rates were slightly higher than 49.7% at 5 years and 43.6% at 10 years, with a mean follow-up period of 69.4 months.

The prognosis of patients with these tumours is poor, with an overall 5-year survival rate of 54.2%, slightly better results than those reported by other studies, in which 5-year survival rates range from 27% to 51% [4,11,12,13,14]. Survival from these tumours is fundamentally conditioned by their high recurrence rate (especially in the form of disseminated disease; in our study, 89.1% of cases), the stage at diagnosis, and the postsurgical residual tumour.

The survival analysis indicated that postmenopausal status, higher FIGO stage (III–IV), and the presence of residual tumours after surgery were significantly associated with a poorer mean survival, in agreement with other studies [2,8,11,18,19,25]. However, no statistically significant differences were observed with respect to OS or with respect to DFS among the different histological subtypes. Finally, although the literature shows that patients with the ESS subtype have the best prognosis [19,23,26], we found a lower OS and a lower DFS in the ESS group, without reaching significance. These differences are probably due to the fact that in our sample 77% of ESS were high-grade.

In this study, we highlighted the differences and similarities of CS and the whole group of other subtypes of uterine sarcomas. CSs appeared more frequently in advanced ages, and consequently, in patients with more associated comorbidities. Unlike the rest of the histological subtypes, a higher percentage of CS cases presented in the form of vaginal bleeding (84.9%, followed by other less frequent subtypes of uterine sarcomas 66.6%) as well as other endometrial carcinomas. Furthermore, CSs tended to have a higher rate of lymph node involvement compared with other subtypes of uterine sarcomas, which may suggest a different biological behavior and would support its reclassification as a high-grade endometrial cancer subtype.

This higher rate of lymph node involvement together with the fact that lymphadenectomy is part of the surgical staging of endometrial carcinoma justify the higher rate of lymphadenectomy in this type of tumours. This is also in line with the higher rates of use of adjuvant treatment (both chemotherapy and radiotherapy) in this tumour type.

However, despite the reclassification of CS as a subtype of endometrial cancer and the systematisation of treatment, CSs still have a poor prognosis and in this study no differences were observed regarding survival between the different histological subtypes.

Among the limitations of the study are those subject to the descriptive nature of the study itself, as well as the long period of patient recruitment, during which new proposals for diagnosis, histological classification, staging, and treatments have been introduced. Another limitation is that the small sample size of low-grade ESS did not allow us to separate them from high-grade ESS, which can make the interpretation of the results difficult. On the other hand, among the strengths, we have already highlighted the high number of patients, from a single centre, resulting from the long recruitment period, allowing us to have a broader view of the entire series and to be able to compare the clinical, histopathological, diagnostic, and therapeutic characteristics of these tumours specifically.

### 4.2. Systematic Review

To our knowledge, this is the first systematic review on the subject. The review grouped a total of 2211 patients with a diagnosis of uterine sarcoma from 19 articles (including ours) published in 16 countries from 2002 to 2021, all with retrospective analyses. There is a growing interest in the topic because more than 68% of the studies have been published in the last decade. We also found a significant increase in articles that excluded CS from 2009 onward in their analysis. Regarding the recruitment period, only one article reported recruiting for less than 10 years, and 31.6% recruited for 20 years or more. Regarding participation in the studies, 73.7% were carried out in single centres.

The average incidence per article/year was 6–7 cases, highlighting the rarity of this type of tumour. The highest incidence rate per year was observed in multicentre studies, highlighting those by Abeler et al. [5] and Wais et al. [10], with annual incidence rates of 13–14 and 21–22 cases, respectively. These higher incidence rates are explained by the multicentre design of the studies.

Based on subtype, we grouped 1106 patients with LMSs, 459 with ESSs, and 416 with CSs. LMS is the most common subtype of uterine sarcoma, representing 45.7% of cases. However, when interpreting these last data, it should be taken into account that 5 of the 19 articles in this systematic review excluded CSs from their analysis and another two did not indicate them as exclusion criteria, but no CSs were part of their sample. The study with the highest % of LMSs was published by Kyriazoglou et al. [4] in 2018, with 83.6% of cases LMS, followed by Wais et al. [10], with 73.2% (both excluded CSs from their study). Among those studies with the highest percentage of LMSs that did not exclude CSs are those by Durnali A et al. [15], with 58%, and Eiriz et al. [11], with 40.3%.

The average age at diagnosis of this type of tumour is 55.8 years, consistent with the observation that this tumour type appears most frequently in individuals between 50 and 70 years of age. Uterine bleeding was the most frequent clinical presentation in 57.3% of patients. The highest percentages of bleeding were reported in the study by Burghaus et al. [8] (81–92%) and in that by Benito et al. [12] (78.6%), neither of which excluded CSs from their analysis. The lowest rates of bleeding were reported in the study by Sait et al. [9] and Gao et al. [3], i.e., 45% and 26.6%, respectively.

It is more common for these tumors to be in the initial stages at diagnosis; 36.3% of the cases presented in advanced stages (III–IV) at diagnosis (ranging between 22.4–53% in the different studies) and reported for the majority of the articles. Based on this systematic review, the recurrence rate was high and close to 42% of the cases, taken in consideration the information from 11 of 19 articles. The study with the highest recurrence rate, 63.4%, was that of Durnali et al. [15], followed by Barquez-Muñoz et al. [18]. Thangappah RBP. [16] presented a recurrence rate much lower than the mean (18.2%); these data should be interpreted with caution since the study had a small sample size (*n* = 30) and there was a total of eight losses to follow-up.

Regarding the OS rate, most studies (13 from 19 articles) collect it at 5 years or less. The average OS rate at 5 years was 41.4%. The highest OS rate was reported by Barquet-Muñoz et al. [18] in a study conducted in Mexico and published in 2018 (61.9%). The 5-year OS rate for our patients was slightly higher than the average (54.2%) but less than Barquet-Muñoz et al. [18]. The lowest OS rate was reported by Kokawa et al. [21] (17.5%) in a study conducted in Japan, which may probably be associated with the fact that a high percentage of the tumors in their sample are diagnosed in advanced stages (51.6%). In general, it could be interesting to study the impact of race on the prognosis of this type of tumor; in our study, it could not be carried out because almost all the patients in our sample were Caucasian (96.8%).

The average DFS rate at 5 years was 36.9%, with the lowest survival rate found in the study by Sait et al. [9] (14%), which could probably be again associated with the fact that a high percentage of the tumors in their sample were diagnosed in advanced stages (53%).

Regarding the limitations of this systematic review, it is of note that most conclusions extracted are based on published data and some heterogeneous criteria including the possibility to exclude/included CS and the absent of data from some variable like DFS rate at 5 or more years. 

## 5. Conclusions

In more than three decades of study, we have found that sarcomas are a rare entity, where CSs account for almost half of all cases; excluding that subtype, LMSs are the most prevalent. Among the different subtypes, we found significant differences between the demographic, clinical, tumour staging, and therapeutic variables as well as in the prognosis related to the survival of these patients, results that are useful for guiding the management of care for patients with uterine sarcomas. Notably, the median OS was 176 months, and the median DFS was 61.8 months, despite not finding significant differences between the subtypes. 

Based on the information in this systematic review, it is of note that women affected by Gyneacological Uterine Sarcoma reported in the last three decades were studied in a Unicenter Hospital, were very infrequent by year, and patients with median age. LMS was the most frequent histologic type, the clinical presentation was bleeding in most of cases, presented in early stage with a recurrence rate, and the OS rates at 5 years were close to 40%. Finally, only a few articles reported information about DFS at 5 or 10 years. Some differences found between articles could be explained by the difficulty in collecting the information from a very rare entity and the heterogeneity and changes in protocols based on the management of these tumours or differences in the inclusion/exclusion criteria considering CS in this group. Therefore, new lines of research are suggested that, together with the information obtained in this work, can help us to propose a multicentre and/or multinational database and identify effective healthcare strategies to affront these tumours. This study aimed to develop strategies for these tumors, in addition to trying to prove that these strategies, since they depend on different variables, can be personalized taking into account each patient’s age, histological type, stage, and risk of recurrence.

## Figures and Tables

**Table 1:** Demographic, clinical characteristics, and tumor characteristics of the patients with uterine sarcoma. Carcinosarcoma were distinguished in the table to make the comparison between the tumours easier. * Incidental diagnosis: incidental finding in the histological study of hysterectomy specimens indicated for suspected benign pathology. Carcinosarcoma were distinguish in the table to make it easier the comparison between the tumours.

Variables	Total (*n* = 161)	LMS (*n* = 51)	ESS (*n* = 31)	CS (*n* = 73)	Others (*n* = 6)	*p*
	Mean ± S.T. n (%)	Mean ± S.T. n (%)	Mean ± S.T. n (%)	Mean ± S.T. n (%)	Mean ± S.T. n (%)	
Age (years) at diagnosis	59.52 ± 13.89	58 ± 27.83	55 ± 20.38	62.75 ± 32.53	62.67 ± 9.91	<0.01
Race						0.789
Caucasian	156 (96.8%)	48 (94.1%)	30 (96.7%)	72 (98.6%)	6 (100%)	
Hispanic	4 (2.48%)	2 (3.92%)	1 (3.22%)	1 (1.36%)	0 (0%)	
Asian	1 (0.62%)	1 (1.96%)	0 (0%)	0 (0%)	0 (0%)	
Comorbidities-General	91 (56.5%)	19 (37.3%)	16 (51.6%)	53 (72.6%)	3 (50%)	0.01
Comorbidities-Obesity	13 (8.07%)	2 (3.92%)	2 (6.45%)	9 (12.3%)	0 (0%)	
Comorbidities-Diabetes	16 (9.93%)	4 (7.84%)	2 (6.45%)	10 (13.6%)	0 (0%)	
Comorbidities-arterial hypertension	54 (33.5%)	12 (23.5%)	11 (35.4%)	30 (41.0%)	1 (16.6%)	
Menopausal status	111 (68.9%)	21 (41.1%)	19 (61.2%)	66 (90.4%)	5 (83.3%)	<0.01
Previous pelvic radiotherapy	3 (1.9%)	0 (0%)	1 (3.2%)	2 (2.7%)	0 (0%)	0.63
Previous use of tamoxifen	8 (4.9%)	0 (0%)	1 (3.2%)	6 (8.2%)	1 (16.7%)	0.097
Clinic at diagnosis						<0.01
Bleeding	103 (63.9%)	18 (35.2%)	19 (61.2%)	62 (84.9%)	4 (66.6%)	
Abdominal mass	18 (11.1%)	7 (13.7%)	3 (9.67%)	7 (9.58%)	1 (16.6%)	
Rapid growth of a fibroid	5 (3.10%)	3 (5.88%)	2 (6.45%)	0 (0%)	0 (0%)	
Incidental diagnosis *	35 (21.7%)	23 (45.0%)	7 (22.5%)	4 (5.47%)	1 (16.6%)	
Stage at diagnosis						0.054
Stage I	86 (53.42%)	34 (66.67%)	17 (54.84%)	33 (45.21%)	2 (33.33%)	
Stage II	15 (9.32%)	2 (3.92%)	2 (6.45%)	11 (15.07%)	0 (0%)	
Stage III	31 (19.25%)	7 (13.73 %)	2 (6.45%)	21 (28.77%)	1 (16.67%)	
Stage IV	19 (11.8%)	3 (5.88%)	9 (29.03%)	5 (6.85%)	2 (33.33%)	
Unknown	10 (6.21%)	5 (9.80%)	1 (3.23%)	3 (4.11%)	1 (16.67%)	

**Table 2:** Treatment characteristics of the patients with uterine sarcoma. Carcinosarcoma were distinguished in the table to make the comparison between the tumours easier.

Variables	Total (*n* = 161)	LMS (*n* = 51)	ESS (*n* = 31)	CS (*n* = 73)	Others (*n* = 6)	*p*
	Mean ± S.T. n (%)	Mean ± S.T. n (%)	Mean ± S.T. n (%)	Mean ± S.T. n (%)	Mean ± S.T. n (%)	
Primary treatment						0.35
Surgery	150 (93.2%)	49 (96.1%)	27 (87.1%)	69 (94.5%)	5 (83.3%)	
Palliative treatment	9 (5.6%)	1 (1.9%)	4 (12.9%)	3 (4.1%)	1 (16.7%)	
Chemotherapy	2 (1.2%)	1 (1.9%)	0 (0%)	1 (1.4%)	0 (0%)	
Diagnosis-surgery interval in days (n = 150)	27.95 ± 39.55	24.88 ± 46.80	20.26 ± 24.29	32.4 ± 38.57	38 ± 41.55	0.473
Surgery time in minutes (n = 43)	120.31 ± 76.79	114.23 ± 74.55	115 ± 53.79	121.16 ± 82.20	210	0.693
Via surgery						0.08
Laparoscopy	20 (13.3%)	3 (6.12%)	2 (7.40%)	13 (18.8%)	2 (40%)	
Laparotomy	112 (74.6%)	39 (79.5%)	23 (85.1%)	48 (69.5%)	2 (40%)	
Unknown	16 (10.6%)	5 (10.2%)	2 (7.40%)	8 (11.5%)	1 (20%)	
Vaginal	2 (1.33%)	2 (4.08%)	0 (0%)	0 (0%)	0 (0%)	
Surgical technique						<0.01
Hysterectomy + bilateral adnexectomy	63 (42%)	31 (63.2%)	13 (48.1%)	16 (23.1%)	4 (80%)	
Hysterectomy + bilateral adnexectomy + pelvic lymphadenectomy	56 (37.3%)	5 (10.2%)	7 (25.9%)	43 (62.3%)	1 (20%)	
Hysterectomy + bilateral adnexectomy + pelvic adn para-aortic lymphadenectomy	1 (0.66%)	0 (0%)	0 (0%)	1 (1.44%)	0 (0%)	
Simple hysterectomy	15 (10%)	9 (18.3%)	5 (18.5%)	1 (1.44%)	0 (0%)	
Debulking	13 (8.66%)	4 (8.16%)	2 (7.40%)	6 (8.69%)	0 (0%)	
Hysterectomy + bilateral adnexectomy + sentinel lymph node biopsy	2 (1.33%)	4 (8.16%)	2 (7.40%)	6 (8.69%)	0 (0%)	
Lymphadenectomy	57 (38%)	5 (10.2%)	7 (25.9%)	44 (63.76%)	1 (20%)	<0.01
Digestive anastomosis	9 (6%)	4 (8.2%)	2 (7.4%)	3 (4.3%)	0 (0%)	0.212
Post-surgical residue						0.239
R0 (Absence)	127 (84.6%)	45 (91.8%)	22 (81.4%)	56 (81.1%)	4 (80%)	
R1 (Microscopic)	3 (2%)	0 (0%)	1 (3.70%)	2 (2.89%)	0 (0%)	
R2 (Macroscopic)	19 (12.6%)	3 (6.12%)	4 (14.8%)	11 (15.9%)	1 (20%)	
Unknown	1 (0.66%)	1 (2.04%)	0 (0%)	0 (0%)	0 (0%)	
No R0	22 (14.6%)	3 (6.12%)	5 (18.5%)	13 (18.8%)	1 (20%)	
Complications	13 (8.6%)	6 (12.2%)	3 (11.1%)	4 (5.7%)	0 (0%)	0.154
Serious III-IV complications (according to CTCA)	7 (4.6%)	3 (6.1%)	1 (3.7%)	3 (4.3%)	0 (0%)	0.129
Reintervention	8 (5.3%)	4 (8.2%)	2 (7.4%)	2 (2.9%)	0 (0%)	0.421
Duration of admission (days) (n = 97)	6.62 ± 7.86	6.04 ± 4.84	6.76 ± 3.91	7.04 ± 10.38	3 ± 1.41	0.866
Perioperative death (30 days)	2 (1.3%)	1 (2%)	1 (3.7%)	0 (0%)	0 (0%)	0.127
In-hospital death (>30 days)	1 (0.7%)	0 (0%)	0 (0%)	1 (1.4%)	0 (0%)	0.568

**Table 3:** Adjuvant therapy. Carcinosarcomas were distinguished in the table to make the comparison between the tumours easier.

Variables	Total (*n* = 161)	LMS (*n* = 51)	ESS (*n* = 31)	CS (*n* = 73)	Others (*n* = 6)	*p*
	Mean ± S.T. n (%)	Mean ± S.T. n (%)	Mean ± S.T. n (%)	Mean ± S.T. n (%)	Mean ± S.T. n (%)	
Adjuvant treatment	86 (53.4%)	19 (37.3%)	12 (38.7%)	55 (75.3%)	0 (0%)	<0.01
Adjuvant chemotherapy	36 (22.4%)	9 (17.6%)	4 (12.9%)	23 (31.5%)	0 (0%)	0.113
Adjuvant radiotherapy	65 (40.4%)	12 (23.5%)	9 (29%)	44 (60.3%)	0 (0%)	<0.01
Adjuvant chemotherapy + adjuvant radiotherapy	15 (9.31%)	2 (3.9%)	1 (3.2%)	12 (16.4%)	0 (0%)	
Type of radiotherapy						<0.01
External	28 (17.3%)	11 (21.5%)	5 (16.1%)	13 (17.8%)	1 (16.6%)	
External + Brachytherapy	36 (22.3%)	1 (1.96%)	5 (16.1%)	31 (42.4%)	0 (0%)	
No adyuvant radiotherapy	97 (60.2%)	39 (76.4%)	21 (67.7%)	29 (39.7%)	5 (83.3%)	

**Table 4:** Characteristics of progression and recurrence among patients with uterine sarcoma. Carcinosarcomas were distinguished in the table to make the comparison between the tumours easier.

Variables	Total (*n* = 161)	LMS (*n* = 51)	ESS (*n* = 31)	CS (*n* = 73)	Others (*n* = 6)	*p*
	Mean ± S.T. n (%)	Mean ± S.T. n (%)	Mean ± S.T. n (%)	Mean ± S.T. n (%)	Mean ± S.T. n (%)	
Tumor progression	27 (16.8%)	6 (11.8%)	8 (25.8%)	12 (16.4%)	1 (16.7%)	0.255
Treatment progression (n = 27)						0.13
Chemotherapy	7 (25.9%)	3 (50%)	1 (12.5%)	3 (25%)	0 (0%)	
Palliative treatment	17 (62.9%)	2 (33.3%)	7 (87.5%)	8 (66.6%)	0 (0%)	
Surgery + Chemotherapy	2 (7.4%)	1 (16.6%)	0 (0%)	1 (8.3%)	0 (0%)	
Unknown	1 (3.7%)	0 (0%)	0 (0%)	0 (0%)	1 (100%)	
Recurrence	73 (45.3%)	27 (52.9%)	13 (41.9%)	31 (42.4%)	2 (33.3%)	0.517
Recurrence type						0.654
Locoregional	8 (10.9%)	2 (7.4%)	3 (23.0%)	3 (9.67%)	0 (0%)	
Single metastases	6 (8.21%)	4 (14.8%)	0 (0%)	2 (6.45%)	0 (0%)	
Multiple metastases	59 (80.8%)	21 (77.7%)	10 (76.9%)	26 (83.8%)	2 (100%)	
Place first recurrence						0.421
Vaginal dome	6 (8.21%)	2 (7.40%)	1 (7.69%)	3 (9.67%)	0 (0%)	
Sarcomatosis	27 (36.9%)	10 (37.0%)	7 (53.8%)	8 (25.8%)	2 (100%)	
Lung	12 (16.4%)	7 (25.9%)	1(7.69%)	4 (12.9%)	0 (0%)	
Ganglion	8 (10.9%)	0 (0%)	1 (7.69%)	7 (22.5%)	0 (0%)	
Multiple metastases	20 (27.3%)	8 (29.6%)	3 (23.0%)	9 (29.0%)	0 (0%)	
Months surgery-recurrence	18.81 ± 20.17	21.18 ± 24.11	9.15 ± 12.42	21.22 ± 18.71	11.22 ± 14.25	0.113
Recurrence treatment						0.656
Surgery	10 (13.6%)	6 (22.2%)	0 (0%)	4 (12.9%)	0 (0%)	
Surgery + chemotherapy	17 (23.2%)	8 (29.6%)	2 (15.3 %)	6 (19.3%)	1 (50%)	
Surgery + radiotherapy	1 (1.36%)	0 (0%)	1 (7.69%)	0 (0%)	0 (0%)	
Surgery + chemotherapy + radiotherapy	1 (1.36%)	0 (0%)	0 (0%)	1 (3.22%)	0 (0%)	
Chemotherapy	17 (23.2%)	8 (29.6%)	2 (15.3%)	7 (22.5%)	0 (0%)	
Radiotherapy	3 (4.10%)	0 (0%)	0 (0%)	3 (9.67%)	0 (0%)	
Hormonetherapy	2 (2.73%)	0 (0%)	1 (7.69%)	1 (3.22%)	0 (0%)	
Palliative treatment	22 (30.1%)	5 (18.5%)	7 (53.8%)	9 (29.0%)	1 (50%)	
Surgery type (recurrence) (n = 29)						0.729
Debulking	17 (58.6%)	8 (57.1%)	2 (66.6%)	6 (54.5%)	1 (100%)	
Single metastases	8 (27.5%)	5 (35.7%)	0 (0%)	3 (27.2%)	0 (0%)	
Others	4 (13.7%)	1 (7.14%)	1 (33.3%)	2 (18.1%)	0 (0%)	
R0 (recurrence)						0.268
Yes	25 (86.2%)	13 (92.8%)	3 (100%)	8 (72.7%)	1 (100%)	
No	4 (13.7%)	1 (7.14%)	0 (0%)	3 (27.2%)	0 (0%)	
Digestive anastomosis (recurrence)	11 (37.9%)	6 (42.8%)	1 (33.3%)	3 (27.2%)	1 (100%)	0.142
Complications (recurrence)	4 (13.7%)	3 (21.4%)	1 (33.3%)	0 (0%)	0 (0%)	0.236
Severe complications (III-IV acording to CTCAE)	2 (6.89%)	2 (14.2%)	0 (0%)	0 (0%)	0 (0%)	0.264

**Table 5:** Most frequent site of involvement in disseminated disease. Carcinosarcomas were distinguished in the table to make the comparison between the tumours easier.

Variables	Total (*n* = 161)	LMS (*n* = 51)	ESS (*n* = 31)	CS (*n* = 73)	Others (*n* = 6)	*p*
	Mean ± S.T. n (%)	Mean ± S.T. n (%)	Mean ± S.T. n (%)	Mean ± S.T. n (%)	Mean ± S.T. n (%)	
Sarcomatosis	66 (40.9%)	24 (47.0%)	18 (58.0%)	22 (30.1%)	2 (33.3%)	0.086
Pulmonary metastases	46 (28.5%)	25 (49.0%)	10 (32.2%)	9 (12.3%)	2 (33.3%)	<0.01
Liver metastases	24 (14.9%)	7 (13.7%)	7 (22.5%)	10 (13.6%)	0 (0%)	0.746

**Table 6:** Long-term follow-up. Carcinosarcomas were distinguished in the table to make the comparison between the tumours easier.

Variables	Total (*n* = 161)	LMS (*n* = 51)	ESS (*n* = 31)	CS (*n* = 73)	Others (*n* = 6)	*p*
	Mean ± S.T. n (%)	Mean ± S.T. n (%)	Mean ± S.T. n (%)	Mean ± S.T. n (%)	Mean ± S.T. n (%)	
Months from surgery to last revision	69.41 ± 72.62	79.77 ± 83.77	43.95 ± 59.95	68.01 ± 63.5	122.86 ± 104.31	0.068
Status last revisión						0.93
Disease free	70 (43.4%)	21 (41.1%)	11 (35.4%)	35 (47.9%)	3 (50%)	
Disseminated disease	11 (6.83%)	3 (5.88%)	2 (6.45%)	5 (6.84%)	1 (16.6%)	
Deceased	75 (46.5%)	25 (49.0%)	17 (54.8%)	31 (42.4%)	2 (33.3%)	
Loss of follow-up	5 (3.10%)	2 (3.92%)	1 (3.22%)	2 (2.73%)	0 (0%)	
Age at death	63.97 ± 13.81	57.27 ± 13.13	60.72 ± 17.56	71.13 ± 8.46	65.88 ± 9.65	0.001
Months recurrence to death	18.9 ± 33.5	27.17 ± 39.9	8.8 ± 10	17.32 ± 34.72	2.32	

**Table 7:** Data on OS and DFS. * Median survival in months (95% CI); ** Mean. Carcinosarcomas were distinguished in the table to make the comparison between the tumours easier.

	All sarcomas (*n* = 161)	LMS (*n* = 51)	ESS (*n* = 31)	CS (*n* = 73)	Others (*n* = 6)	Log Rank (*p* Value)
OS *	176.04 (106.3–245.74)	56.1 (0.174.5)	16.05 (0–40.89)	141.56 **	186.27 **	0.283
5 year OS (%)	54.2	49.5	44.7	61.5	80	
10 year OS (%)	46.2	46.6	44.7	51.8	80	
DFS *	61.86 (34.96–88.75)	52.38 (25.37–79.38)	16.05 (0–41.40)	64.41 (31.44–97.37)	232.05	0.302
DFS 5 year (%)	49.7	46.8	38.1	55.9	80	
DFS 10 year (%)	43.6	40.7	38.1	44.7	80	

**Table 8:** Main variables collected in the systematic review. NR: no reported. * 8 patient losses in an n of 30.

Country	Period	Uni/Multricentric	n	Incidence Year	Excludes CS	LMS	LMS %	SEE	CS	Mean Age	Most Frecuent Clinic at Diagnosis	Advances Stages III-IV (%)	Recurrence (%)	OS 5 Year (%)	OS 10 Year (%)	DFS 5 Year (%)	DFS 10 Year (%)
Czech Republic	1990–1999	Unicentric	49	4.9	No	17	34.7	11	21	55.8	NR	22.4	NR	26.5	NR	24.5	NR
Japan	1990–2003	Multicentric	97	6.9	No	36	37.1	15	46	59	NR	51.6	NR	17.5	NR	NR	NR
Finland	1990–2001	Unicentric	100	8.3	No	39	39	21	40	60	NR	39	50	51	38	NR	NR
Korea	1989–2007	Unicentric	127	6.7	No	46	36.2	37	44	50	59.1% bleeding	29.13	37	59	48	53	30
Spain	1990–2006	Unicentric	89	5.2	No	20	22.5	18	43	62	78.6% bleeding	30.3	35	45	39	61	55
Norway	1970–2000	Multicentric	419	13.5	Yes	235	56.1	85	0	57	NR	NR	NR	NR	NR	NR	NR
Turkey	2000–2010	Multicentric	93	8.5	No	54	58.1	9	25	53	54.7% bleeding	36.56	63.4	49.4	NR	16.5	NR
Mexico	1983–2009	Unicentric	77	2.9	Yes	53	68.8	11	0	52	46.7% bleeding	29.87	42.85	NR	NR	NR	NR
China	1988–2007	Unicentric	80	4.2	No	22	27.5	38	18	57	45% bleeding	50	47.5	40	NR	NR	NR
Saudi Arabia	2000–2012	Unicentric	36	2.8	No	7	19.4	0	21	58	NR	53	22	18	NR	14	NR
Germany	1984–2013	Unicentric	143	4.8	No	57	39.9	23	49	58	81–92% bleeding	27.97	42	51	NR	NR	NR
USA	2001–2014	Multicentric	302	21.6	Yes	221	73.2	81	0	55	74.5% bleeding	29.8	NR	NR	NR	NR	NR
Greece	2001–2016	Unicentric	61	3.8	NR	51	83.6	3	0	53	NR	49.2	NR	NR	NR	NR	NR
India	2009–2015	Unicentric	30	4.3	No	10	33.3	6	9	NR	36.66% abdominal pain and mass	30	18.18*	30	NR	NR	NR
Mexico	2000–2014	Unicentric	73	4.9	Yes	36	49.3	9	0	48	45.2% bleeding	34.24	58.9	61.9	NR	39.7	NR
Italy	1996–2016	Multicentric	195	9.3	NR	116	59.5	48	0	56	49.7% bleeding	29.8	NR	NR	NR	NR	NR
USA	1996–2015	Unicentric	17	0.9	Yes	10	58.8	5	0	50	NR	NR	NR	NR	NR	NR	NR
Portugal	2003–2017	Unicentric	62	4.1	No	25	40.3	8	27	62	NR	43.54	NR	38.7	NR	NR	NR
Spain	1985–2020	Unicentric	161	4.6	No	51	31.7	31	73	59	63.9% bleeding	31.05	45.3	54.2	46.2	49.7	43.6

## Data Availability

The data used to support the findings of the present study are available from the corresponding author upon request.
